# Coherent Optical Transduction of Suspended Microcapillary Resonators for Multi-Parameter Sensing Applications

**DOI:** 10.3390/s19235069

**Published:** 2019-11-20

**Authors:** Alberto Martín-Pérez, Daniel Ramos, Javier Tamayo, Montserrat Calleja

**Affiliations:** Bionanomechanics Lab, Instituto de Micro y Nanotecnología, IMN-CNM (CSIC), Isaac Newton 8 (PTM), E-28760 Tres Cantos, Madrid, Spain; alberto.martin@csic.es (A.M.-P.); jtamayo@imm.cnm.csic.es (J.T.); montserrat.calleja@csic.es (M.C.)

**Keywords:** microcapillary, transparent resonators, interferometry, optomechanics

## Abstract

Characterization of micro and nanoparticle mass has become increasingly relevant in a wide range of fields, from materials science to drug development. The real-time analysis of complex mixtures in liquids demands very high mass sensitivity and high throughput. One of the most promising approaches for real-time measurements in liquid, with an excellent mass sensitivity, is the use of suspended microchannel resonators, where a carrier liquid containing the analytes flows through a nanomechanical resonator while tracking its resonance frequency shift. To this end, an extremely sensitive mechanical displacement technique is necessary. Here, we have developed an optomechanical transduction technique to enhance the mechanical displacement sensitivity of optically transparent hollow nanomechanical resonators. The capillaries have been fabricated by using a thermal stretching technique, which allows to accurately control the final dimensions of the device. We have experimentally demonstrated the light coupling into the fused silica capillary walls and how the evanescent light coming out from the silica interferes with the surrounding electromagnetic field distribution, a standing wave sustained by the incident laser and the reflected power from the substrate, modulating the reflectivity. The enhancement of the displacement sensitivity due to this interferometric modulation (two orders of magnitude better than compared with previous accomplishments) has been theoretically predicted and experimentally demonstrated.

## 1. Introduction

None of what nanotechnology has achieved in recent years would have been possible without the development of the current advanced nanofabrication techniques, gathered together in a nanofabrication toolbox, allowing for the fabrication of smaller and smaller devices. Nowadays, we can use these nanodevices for many different applications, where sensing applications especially benefit from the unprecedented sensitivities achieved by taking advantage of the new properties at the nano scale [1,2]. In this sense, nanomechanical sensors have been demonstrated as one of the most promising devices. The applications of nanomechanical sensors include biology [3,4], chemistry [5], or even fundamental physics [6]. Ever-increasing control of the nanotechnology toolbox allows for the shrinking of device sizes to the nano scale, which increases the sensitivity and functionality of the devices. However, the measurement of the mechanical displacement of the device ends up hampered by the size itself. These systems include all variations of electrical methods, either capacitive or resistive methods [7], and mechanical readout systems [8], among others. However, probably the most promising in terms of sensitivity and versatility are optical readout methods [9]. The use of optical methods for readouts in nanotechnology, more precisely in nanomechanics, dates back to the beginning of nanotechnology itself, with the invention of atomic force microscopy [10] (AFM), in which the optical beam deflection technique was developed. The sensitivity of optical methods for the measurement of nanometric, or sub-nanometric, displacement of devices can be increased by using the properties of coherent light by means of interferometry. Today, an interferometer is used for the development of the most sensitive displacement sensor in mankind’s history—the Laser Interferometer Gravitational-Wave Observatory (LIGO) experiment, which allowed for the measurement of gravitational waves [11]. However, the optical methods can be used not only for measurement systems, but also for actuation [12,13]. The interplay between photons and phonons ends with a whole new concept, optomechanics [14,15], where the bidirectional energy transfer in between the light and mechanics allows for the active changing of mechanical properties (or optical properties) by the optical field (or by the mechanical displacement).

Optomechanical sensors have special needs in terms of design and fabrication [16]. The main limiting factor in design is that we have to increase the interaction between the photons and the phonons, i.e., the light and the mechanical device. The canonical way to enhance this interaction is by placing the nanomechanical resonator inside an external optical cavity, or even by using an optical cavity sustained by the nanomechanical resonator itself, which is referred to as the optomechanical cavity [17,18]. Originally designed for pursuing the quantum ground state of a mesoscopic object [19,20], optomechanical cavities can be found in multiple forms, such as photonic crystal cavities [21], semiconductor toroids or disks [22,23], and Fabry–Perot cavities [24]. However, the optomechanical phenomena is not strictly restricted to a cavity, e.g., optomechanical amplification and cooling have been demonstrated by using the confined electromagnetic modes inside a subwavelength dielectric structure [25]. In this sense, in order to truly confine the electro-magnetic field at the nano scale, we can use plasmonic devices [26,27], where the excitation of a localized surface plasmon resonance is translated into an absorbed optical power, becoming a hotspot and consequently shifting the mechanical resonance frequency of the nanomechanical sensor. Experimental schemes in which a plasmonic device is used as a sensor have been used for many years, especially in biosensing due to its biocompatibility in terms of materials (usually it requires noble metals) and environmental conditions, as it works perfectly in liquids [28]. Optomechanics in liquids [29] is an emerging field that deals with the external damping imposed by the dragging force exerted by a liquid when an immersed object (the nanomechanical resonator surrounded by the liquid) moves. There are two ways to overcome this issue: to use the liquid as a waveguide, including it in the optical cavity [30], or developing a hollow mechanical resonator [31,32] in which the liquid flows inside the structure while the structure vibrates in a vacuum or gaseous atmosphere [33,34]. In this last case, it is possible to perform mass measurements by tracking the resonance frequency of the flexural modes [31].

In this work, we have developed a novel technique for fabricating a hollow optomechanical resonator. This resonator is based in an optically transparent glass capillary [35,36] which interacts with the electromagnetic field by means of a homemade interferometric system. The use of a coherent light to measure the mechanical displacement opens the door to an optomechanical amplification of movement, boosting the mechanical quality factor, and consequently increasing the frequency resolution by decreasing the noise. However, as we will show in this study, the electromagnetic field distribution, which could be used to exert an optical feedback force, depends on the position along the suspended capillary as a consequence of its geometry, which allows for the control of the amplitude signal by simply displacing the sample. First of all, we are going to theoretically show and experimentally demonstrate the coupling in between the evanescent field emerging from the silica surface with the surrounding electromagnetic field. The optomechanical displacement sensitivity (defined as variation of reflectivity as a function of displacement) will be subsequently theoretically derived and experimentally demonstrated, showing an enhancement when compared with a non-coherent transduction scheme of two orders of magnitude, from 3 × 10^−4^ µm^−1^ to 2 × 10^−2^ µm^−1^. Finally, depending on the position along the capillary length, we characterize the frequency noise of the system, demonstrating the expected modulation.

## 2. Results and Discussion

**Figure 1:**
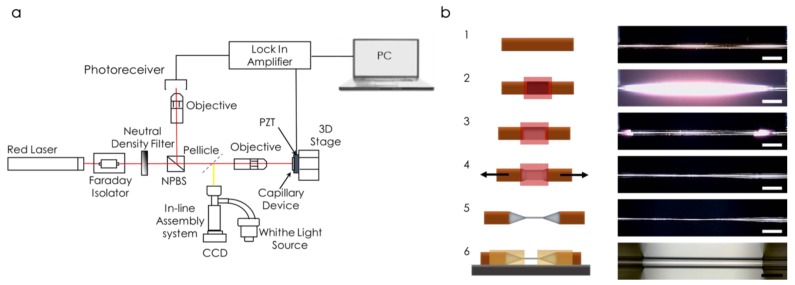
(**a**) Schematics of the experimental setup. (**b**) Thermal stretching process. Step 1: original capillary. Step 2: initial heating. Step 3: polyimide removal. Step 4: axial pulling. Step 5: final capillary. Step 6: Photolithography of the supporting pads. The optical micrographs show the whole process, the white scale bars are 1 mm, whereas the black scale bar is 100 µm.

The mechanical displacement of the capillary was optically detected by using a homemade interferometer [37] (Figure 1a). In this system, we focused a laser beam (5 mW, 632.8 nm, Research Electro-Optics, Inc.), after adjusting the intensity by means of a neutral density filter, through a 50:50 non-polarizing beam splitter (NPBS) and a 20X 0.42 NA long working distance objective (Mitutoyo) on the middle of the suspended region of the capillary (beam waist ~10 μm). The low numerical aperture objective focused the laser on the sample, and subsequently collected the reflected power. The intensity of the recombined light was measured by a photodetector (model PDA 10A-EC, Thorlabs, Inc., NJ, USA). The voltage signal provided by the photodetector was split into its AC and DC components. The AC signal contained the information about the displacement of the resonator, while the DC revealed information about the power reflected by the capillary. Both signals were analyzed by a locking amplifier (HF2LI-PLL, Zurich Instruments, Zurich, Switzerland) whose output voltage signal was used as driving signal for a piezo shaker to excite the mechanical modes of the capillary.

In order to enhance the optomechanical coupling, it was necessary to fabricate an optically transparent hollow resonator (Figure 1b). Therefore, starting with a commercially available glass capillary (Polymicro Technologies, TSP250350) with a 350 µm outer diameter and a 250 µm inner diameter covered with a 20 µm capping layer of polyimide (step 1 in Figure 1b), we developed a stretching process by heating up the capillary (step 2 in Figure 1b) while applying a controlled axial stress (step 4 in Figure 1b), which set the final dimeter of the device within a 1 μm precision. The final outer diameter was set to 56 µm and the inner diameter to 41 µm (step 5 in Figure 1b). The local heating was applied by a controlled flame, which removed the capping layer by pyrolysis (step 3 in Figure 1b), revealing the transparent glass (fused silica) capillary. Once the polyimide layer was removed, we applied an axial by pulling it under the microscope until the desired size was reached. We subsequently patterned the clamping SU8 pads by a standard cleanroom photolithography process: SU8 spin coating, ultraviolet light exposure using a mask, and developing (step 6 in Figure 1b). The whole procedure resulted in a suspended double clamped transparent silica capillary (optical micrograph in Figure 1b). 

**Figure 2:**
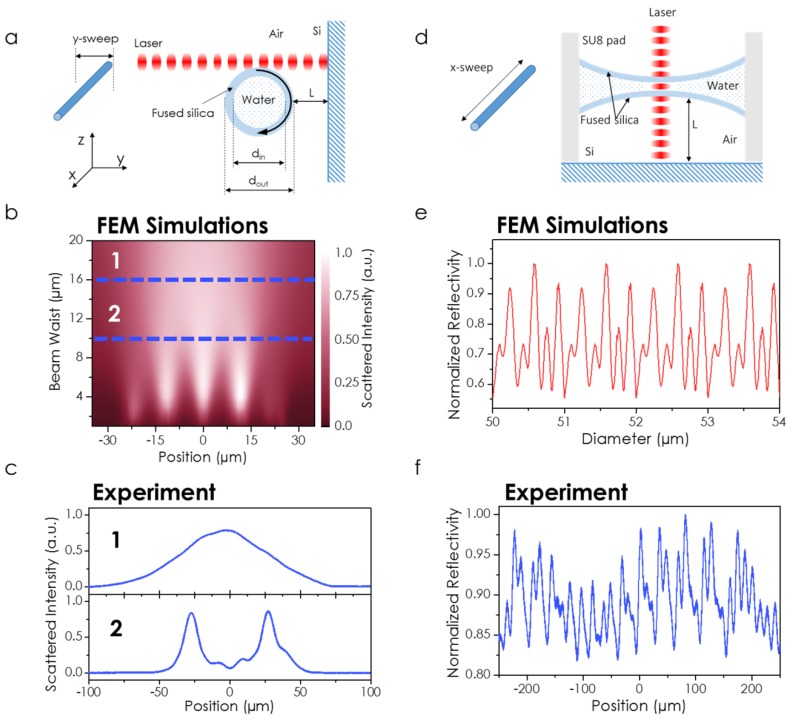
(**a**) Schematics of transversal sweep (y-sweep). (**b**) Simulations by using Finite Element Method (FEM) of the scattered intensity while varying the beam waist for different relative positions of the capillary and the laser beam (y-sweep). (**c**) Experimental demonstration of the scattered intensity for y-sweeps at different beam waists. (**d**) Schematics of longitudinal sweep (x-sweep). (**e**) FEM simulations of the scattered intensity as a function of the outer diameter of the capillary. (**f**) Experimental demonstration of the scattered light modulations for an x-sweep.

The use of super long working distance microscope objectives allows for the confinement of the electromagnetic field in volumes smaller than the actual size of the capillary wall thickness. Therefore, harnessing the low refractive index contrast, it is possible to couple light inside the fused silica walls. This light evanescently interacts with the standing wave generated by the interference of the reflected power coming out from the silicon substrate underneath the incident light. Thus, the evanescence interaction between the light trapped in the fused silica and the surrounding electromagnetic field depends on the capillary-substrate distance. The thermal stretching process described in the fabrication procedure predefines this distance. Please note that the distance with the substrate depends on the initial/final outer diameter ratio, which varies along the axial direction due to the thermal process. This fabrication aspect opens the door for a double-side optical cavity probing. We have mounted the capillary on a 3D piezo-controlled positioner; therefore, we can scan the laser spot across the sample. Depending on the scanning direction, it is possible to probe both the wall thickness optical cavity at one fixed capillary–substrate distance, or the interference pattern generated by the light bouncing inside the length-varying optical cavity sustained by the capillary and the silicon substrate underneath (Figure 2). Figure 2a shows a schematic depiction of the transversal scanning (along the *y*-axis according to the designed coordinates). With this configuration, we are going to probe the fused silica trapping while keeping the distance to the substrate and the diameter constant. In order to demonstrate the optical cavity, we are going to change the laser beam waist (red laser, wavelength λ=632.8 nm) from 1 µm up to the size of the capillary for each 70 µm y-sweep. First of all, we are going to perform a simulated study by using the Finite Element Method (FEM, Comsol Multiphysics). In Figure 2b, we observe the transition from a laser waist comparable to the capillary diameter up to a focused laser spot of 1 µm. We have calculated the integral of the norm of the electric field at the far field over a solid angle of 30 degrees to mimic the experimental conditions. This is equivalent to simulating the measured scattered light as a function of the y-position along the cross section of the capillary, as shown in Figure 2a. When the size of the laser spot is comparable to the capillary diameter, the scattered light shows a gaussian profile (dashed line labeled as 1 in Figure 2b). However, as the size of the illuminating spot decreases, the smaller features corresponding to the different interlayer boundaries come out, revealing different maxima at a beam waist of 10 µm (dashed line labeled as 2). This behavior is experimentally demonstrated in Figure 2c.

The Fabry–Perot cavity sustained by the suspended capillary and the silicon substrate is probed in Figure 2d, where we show the schematics of the sweeping direction. The thermal stretching process ends up with a hyperbolic profile in the suspended capillary. We have measured a maximum variation of less than 1% in the actual thickness along the whole suspended area, which is about 4 µm along the 500 µm length of the capillary. By considering the laser wavelength, this variation is large enough to cause interferometric modulation in the measured reflectivity. This has been simulated in Figure 2e, where we have parametrized the outer diameter of the fused silica capillary, accordingly linking the inner diameter to the 60% of the outer to mimic the experimental conditions. The modulation observed in the simulations has also been experimentally demonstrated, and is shown in Figure 2f.

Once we have characterized the reflectivity in our system, it is possible to define the mechanical displacement sensitivity, which is given by the derivative of the reflectivity [38]. Therefore, the expected signal for the amplitude of the oscillations as a function of the spatial coordinate x, hereinafter referred as “experimental mode shape”, is derived by multiplying the sensitivity by the eigenvector $\psi_{n}\left(x\right)$, or “theoretical mode profile”,

(1) $$A=\left|\frac{\partial R\left(x\right)}{\partial x}\right|\times \psi_{n}\left(x\right)$$

where R(x) is the reflectivity, which depends on the position, *x* is the coordinate along the long axis of the capillary, and $\psi_{n}\left(x\right)$ is the profile of the *n*-th mechanical mode,

(2) $$\psi_{n}\left(x\right)=cos\left(\frac{x\;\beta_{n}}{L}\right)-cosh\left(\frac{x\;\beta_{n}}{L}\right)+\frac{cos\beta_{n}-cosh\beta_{n}}{sin\beta_{n}-sinh\beta_{n}}\left(sinh\left(\frac{x\;\beta_{n}}{L}\right)-sin\left(\frac{x\;\beta_{n}}{L}\right)\right)$$

L being the capillary length, the first eigenvalues are, $\beta_{n}=4.7300,\;7.8532,\;10.9956,\;14.1372,\;\ldots$ [39].

**Figure 3:**
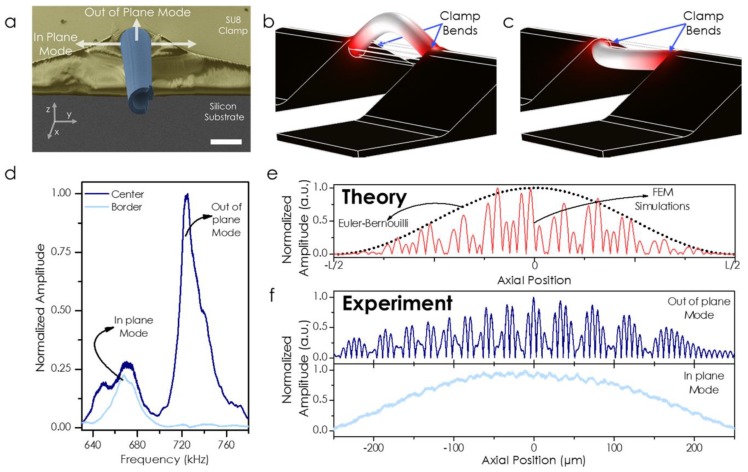
(**a**) Scanning electron microscope image (in false color) of the cross section of the fabricated device. The SU8 pads (in yellow) are not isotropic; therefore, there is a difference in the effective stiffness of the two orthogonal directions, labeled in the images as in-plane and out-of-plane modes. The scale bar is 50 µm. (**b**) Simulations by using Finite Element Method (FEM) of the out-of-plane mode. (**c**) Simulations by using Finite Element Method (FEM) of the in-plane mode. (**d**) Experimental measurement of the mechanical spectrum at two different positions: at the capillary axis (dark blue curve) and at one side (light blue curve). (**e**) Expected mechanical amplitude mode profile for the fundamental mechanical mode, out-of-plane mode. (**f**) Experimental demonstration of the modulation in the amplitude mode profile for the out-of-plane mode (dark blue curve) and the in-plane mode (light blue curve).

Figure 3a shows a scanning electron microscope image of the cross section of the capillary, the scale bar is 50 µm. As can be seen from the image, the SU8 pads are not homogeneous blocks, the photolithographic procedure leaves a 20 µm homogeneous thick polymer layer far from the capillary (as expected in a flat substrate) but this thickness is continuously increased in the y direction, reaching its maximal value at the position of the capillary axis. This inhomogeneous clamping defines two directions for the mechanical modes: in-plane modes (softer clamping) and out-of-plane modes (harder clamping), marked as white arrows in Figure 3a. Both modes are simulated in Figure 3b,c, showing different resonance frequencies due to the difference in the effective spring constant of both directions. It is possible to experimentally demonstrate the existence of both modes by optically measuring the mechanical spectrum at the middle point of the capillary (dark blue line in Figure 3d). However, if the measurement is performed at one side of the capillary (by simply displacing the laser spot 19 µm from the middle point in the y direction), we are only sensitive to the in-plane mode (light blue curve in Figure 3d). Please note, in principle, the system is only sensitive to out-of-plane displacements; therefore, the sensitivity of the in-plane mode mainly comes from the misalignment respect to the substrate, but we cannot neglect the fact that the reflection on a moving cylindrical surface also gives a modulation in the reflected power. From the reflectivity simulation of Figure 2e it is possible to extract the expected mode shape as described in Equation (1). The expected mode shape, Figure 3e, ends up with a modulated amplitude in the mode profile. This expected mode shape is experimentally demonstrated in Figure 3f, where we show the interferometric measurement of the out-of-plane mode (dark blue curve) and the in-plane mode measured 19 µm away from the capillary axis in the y direction (light blue curve). At this position, the interference pattern is vanished because we are only collecting the scattered light.

**Figure 4:**
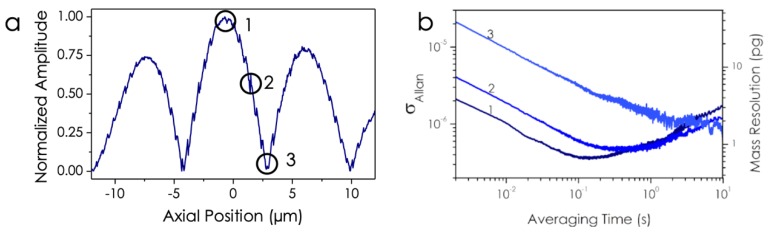
(**a**) Normalized amplitude of the out-of-plane mechanical mode as a function of the measurement position along the axial coordinate. (**b**) Experimental measurement of Allan deviation at three different points, maximum of amplitude, line 1, minimum of amplitude, line 3, and an intermediate point, line 2.

The figure of merit of our optomechanical transduction system is the frequency stability. The way to characterize the noise level of our readout system is to calculate the Allan deviation, $\sigma_{Allan}\left(\tau \right)=\sqrt{\sigma_{Allan}{}^{2}\left(\tau \right)}$, where $\sigma_{Allan}{}^{2}\left(\tau \right)$ is defined as the Allan variance and is calculated from the average of frequency samples measured in a temporal integration time τ, σAllan2(τ)=12〈(f¯(t+1)−f¯(t))2〉. The higher the signal-to-noise ratio, the lower the frequency noise measured [40]. Therefore, by using our optomechanically enhanced displacement sensitivity, we should be able to reduce the frequency noise. Figure 4a shows the mode profile of the out-of-plane mechanical mode for an axial scanning position of 25 µm. As it was described above, the mode amplitude is modulated along its axial coordinate due to the continuous variation of the diameter. By simply displacing the laser spot by 2.5 µm from the center position, the maximum oscillation amplitude of the out-of-plane mode becomes a minimum. This difference is also observed in the Allan deviation measurement (Figure 4b). It is possible to decrease the frequency noise by one order of magnitude from the measurement point labeled as 1 in Figure 4a to the measurement point labeled as 3. The Allan deviation reaches a value of $2\;\times 10^{-7}$ for an integration time of 100 ms at measurement point 1, one order of magnitude lower than previous works with non-coherent optical transduction method [35,36]. This Allan deviation can be converted into mass resolution by considering that the frequency shift produced by a punctual particle (as described in Equation (3)), obtaining a mass resolution of 600 fg for an integration time of 100 ms at measurement point 1,

(3) $$\frac{m_{b}}{m_{0}}=\left(1-\frac{f_{0}}{f_{0}+\Delta f}\right)\psi_{1}\left(L/2\right)$$

where *m_b_* is the buoyant mass of the particle, *m*_0_ the mass of the resonator (including the liquid), *f*_0_ the initial resonance frequency, and Δf the frequency shift caused by the particle.

## 3. Conclusions

In conclusion, we have developed an optomechanical transduction scheme for optically transparent fused silica capillaries. The capillaries have been fabricated by using a thermal stretching technique, which allows the controlling of the final dimensions of the device accurate to 1 µm. These hollow nanomechanical resonators could be used for in-flow mass sensing, where a liquid flows inside the capillary, carrying analytes to be detected. Therefore, a very sensitive mechanical displacement technique is necessary. Here, we show how the light coupled into the fused silica capillary walls interact with the surrounding electromagnetic field distribution, a standing wave sustained by the interference of the incoming illuminating light and the reflection from the silicon substrate. The interference of the evanescent field from the fused silica and the standing wave ends up with a modulation of the sensitivity to mechanical displacement depending on the substrate–capillary separation. The sensitivity of our optomechanical transduction technique is good enough to account for a deviation of less than 1% of the cylindrical shape of our capillary due to the fabrication process. This technique opens the door for a plethora of novel sensing applications for measuring not only the mass of analytes flowing inside vibrating capillaries, but also their optical properties.
